# Clinician perception of pathological narcissism in females: a vignette-based study

**DOI:** 10.3389/fpsyg.2023.1090746

**Published:** 2023-04-20

**Authors:** Ava Green, Rory MacLean, Kathy Charles

**Affiliations:** ^1^Department of Psychology, City, University of London, London, United Kingdom; ^2^Department of Psychology, Edinburgh Napier University, Edinburgh, United Kingdom; ^3^Centre for Academic Development and Quality, Nottingham Trent University, Nottingham, United Kingdom

**Keywords:** female narcissism, pathological narcissism, vulnerable narcissism, gender differences, gender bias, diagnosis, treatment

## Abstract

The DSM-5 reports that up to 75% of those diagnosed with Narcissistic Personality Disorder (NPD) are males, which denotes that narcissism is a clinical phenomenon that operates differently in men and women. Vulnerable narcissism, which tends to be more prevalent in females and is currently under-appreciated in the DSM-5, may be diagnosed as other “vulnerable” disorders (e.g., Borderline Personality Disorder; BPD). The current study investigated gender differences in clinicians’ perceptions of narcissistic pathology. Adopting an online vignette-based study, clinicians (*N* = 108; 79 females) read clinical case vignettes of hypothetical patients and provided diagnostic ratings of existing personality disorders. Clinicians’ diagnostic ratings of NPD were concurrent with the vignette containing grandiose narcissism symptoms, irrespective of patient gender. However, when presented with a vulnerable narcissism vignette, clinicians were significantly more likely to attribute a BPD diagnosis in female patients, compared to male patients. Clinicians with a psychodynamic approach and more experience in practice were also more likely to label vulnerable narcissism symptoms as NPD, compared to those with a CBT approach and less experience in practice. The clinical implications of these results support the shift toward assessing personality dysfunction based on dimensional trait domains.

## Introduction

The issue of gender bias across the Diagnostic and Statistical Manual of Mental Disorders (DSM-5) personality disorder criteria in general, and Narcissistic Personality Disorder (NPD) in particular, is controversial and has been widely debated. Compared to other diagnostic manuals that integrates grandiose and vulnerable expressions of NPD (e.g., the Psychodynamic Diagnostic Manual-Second Edition; PDM-2; Lingiardi and McWilliams, 2017), NPD as codified in the DSM-5 predominantly captures overt grandiosity, including symptoms such as interpersonal exploitation, entitlement, exhibitionism, lack of empathy, and self-serving fantasies of omnipotence. The DSM-5 reports that up to 75% of those diagnosed with NPD are males (American Psychiatric Association, 2013), which suggests that the representation of narcissism (NPD DSM-5) may only apply marginally to females, due to its overemphasis on capturing grandiose themes at the expense of vulnerable variants of the disorder (Levy et al., 2011). Vulnerable narcissism includes elements of shyness, hypersensitivity, rumination, shame, and low self-esteem (Pincus et al., 2009). The gender bias in the conceptualization of narcissism was recognized by early theorists who contested that clinical observations made by Kernberg (1975) and Kohut (1977) have emerged from patriarchal and phallocentric narratives that overemphasize masculinity and the male syndrome, whereas feminine voices are demoted (Akhtar and Thomson, 1982; Philipson, 1985; Richman and Flaherty, 1988).

For instance, Philipson (1985) stated that Kernberg’s (1975) and Kohut’s (1977) discoveries derived from 29 clinical case studies of patients exhibiting NPD traits, of which only five cases depicted women. The disproportionate sample of male patients was noteworthy due to the clinical population consisting predominantly of female psychiatric patients (Philipson, 1985). In other words, such figures preclude the assumption that the gender ratio is an artifact of sampling bias in the psychiatric setting, and, in turn, support the contention that narcissistic pathology as captured in the DSM-5 is understood primarily, if not exclusively, through the perspective of males. Indeed, grandiose features of narcissism have been closely linked to male socialization characteristics, including displays of physical aggression, authority, and an excessive need for power and status (Corry et al., 2008; Grijalva et al., 2014). Whilst females are less likely to exhibit overt “stereotypical” narcissistic features, their expression of narcissistic pathology may resemble more feminine qualities associated with vulnerable features, such as shame, low self-esteem, and inhibition (Green et al., 2021). Although some research reports no gender differences on vulnerable narcissism measures (e.g., Grijalva et al., 2014), other reviews report a higher female preponderance (see Green et al., 2021), albeit the effect size of these gender disparities range from small to medium (e.g., Pincus et al., 2009; Wright et al., 2010).

The extent to which the construct and prevalence of NPD is, in fact, gender-biased has significant implications for the diagnosis and treatment across gender. Clinical studies have found that clinicians may be more likely to treat patients who present narcissistic vulnerability, compared to those who present narcissistic grandiosity. This is because of the increased compliance with treatment associated with patients presenting narcissistic vulnerability, compared to patients presenting narcissistic grandiosity (Pincus et al., 2009; Ellison et al., 2013). These findings, however, convey a mismatch between the presentation of grandiose narcissism as captured by the DSM, which tends to be more prevalent in men, and vulnerable narcissism, which is currently barely considered by the DSM and tends to be more prevalent in women (Green et al., 2021). This is particularly concerning in light of the potential misdiagnosis of vulnerable narcissism, given its overlap with BPD (Miller et al., 2010; Euler et al., 2018) and avoidant and dependent personality disorders (Dickinson and Pincus, 2003; Miller et al., 2014).

### Gender bias in clinical judgment of narcissistic personality disorder

The clinical and empirical literature has consistently established a significant link between the male gender and NPD (Lindsay et al., 2000; Karterud et al., 2011; Hoertel et al., 2018). These findings are commonly grounded in the assumption that the criteria of NPD are gender-biased, where males and females are traditionally considered to manifest the disorder differently due to gender-related symptomatology. Independent of any actual differences in classifications of personality disorders (PDs) between males and females, misdiagnoses may partly contribute to the differential prevalence rates of PDs observed in the DSM-5 (Schulte and Habel, 2018). Research has argued that vulnerable narcissism may be overlooked or misdiagnosed as BPD in female patients particularly, whereas males are more prone to be diagnosed with NPD due to their overt grandiose presentation of narcissism (Euler et al., 2018). This is significant as females are more prone to seek treatment than males (Skodol and Bender, 2003), and clinicians are more likely to treat patients with NPD when they are in a vulnerable state (Ellison et al., 2013). These findings reflect the preponderance of females diagnosed with BPD in clinical settings, as the latter does not resemble the balanced gender ratio found in epidemiological cohorts (Paris et al., 2013).

Providing further support for the above theorizations, a research study by Anderson et al. (2001) found that clinicians diagnosed narcissistic and antisocial PDs more frequently in men, whereas women were more likely to be diagnosed with borderline, dependent, and histrionic PDs. The authors noted that clinicians did not consider the diagnostic criteria to be more (or less) maladaptive or pathological for a man than for a woman. Rather, clinicians perceived men to be more physically aggressive and more likely to exhibit a grandiose self-image than women. This invites the possibility that the differential prevalence rates in diagnoses may be partly due to gender stereotyping. It must be noted, however, that longstanding gender differences have been shown to be rooted in biological differences where men are generally more physically aggressive than women (e.g., Skodol and Bender, 2003; Schulte and Habel, 2018).

Interestingly, research has revealed that sex bias^1^ in diagnosis may, in part, occur due to the ambiguity of the case. Braamhorst et al. (2015) presented trainee clinicians with hypothetical case vignettes containing an ambiguous case (which contained subthreshold features of both NPD and BPD) and a non-ambiguous case (which contained subthreshold features of either NPD or BPD). The authors distinguished two underlying mechanisms for sex bias: gender stereotyping and actual base rate variations (differences observed in males and females due to factors other than gender stereotypes). Results showed that there was no effect of sex of patient for non-ambiguous vignettes; however, when the case was ambiguous, participants diagnosed BPD more often in females than in males, and NPD more often in males than in females. The authors concluded that when there is ambiguity in the classification of PD, sex bias is present and more likely to be influenced by base-rate variation than gender stereotyping. An acknowledged limitation and suggestion for future research pertained to the inclusion of participant characteristics (e.g., years of experience, type of psychotherapy training) for a finer-grained analysis.

### Treatment of narcissistic personality disorder

The descriptive characteristics of narcissism and diagnostic criteria that best exemplify the construct have been much debated. These disparities have been poorly calibrated across the psychiatry, clinical, and social/personality literature, reflecting enduring disagreement among clinicians and experts with regard to the central features of narcissism (Green et al., 2021). For instance, research from the social/personality literature questions the notion that narcissistic grandiosity and vulnerability “co-exist” (e.g., Miller et al., 2018), whereas the clinical literature suggests narcissistic individuals oscillate between the two dimensions (Cain et al., 2008). Crucially, although both experts in the social/personality field and clinicians generally believe that the grandiose features are central to narcissism, clinicians also consider concurrent vulnerability to be a defining feature of the construct (Ackerman et al., 2017). Despite these differences in opinions, Ackerman et al. (2017) found that clinicians have little to no consensus in their views regarding the centrality of vulnerable characteristics in NPD, perhaps reflecting different therapy orientations shaping clinicians’ understanding of narcissism and the related central pathognomonic features (i.e., characteristics of a particular condition).

The efficacy of psychotherapeutic approaches and evidenced-based treatments for NPD is limited (Caligor et al., 2015). It has been argued that, in the absence of empirically supported treatments for NPD, it is common practice to utilize other effective treatments from “near-neighbor” disorders, such as BPD (Kealy et al., 2017). Indeed, researchers have posited that treatments designed explicitly for BPD patients, such as dialectical behavioral therapy, might be usefully employed for individuals with vulnerable narcissism, given their similar nomological networks (Kaufman et al., 2018). In the case of individuals with grandiose narcissism, researchers have argued that these individuals are likely to require different therapeutic approaches compared to their vulnerable counterparts (Ogrodniczuk et al., 2009). However, in a study gathering clinicians’ preferred therapy for patients with NPD, grandiose and vulnerable presentations of narcissism were associated with the same treatment approach (Kealy et al., 2017).

The lack of clarity regarding preferred treatment choices for patients with grandiose and vulnerable expressions on the one hand, and research which demonstrates that clinicians’ therapeutic orientations can significantly affect their diagnostic judgments (Woodward et al., 2009) on the other hand, suggests that exploring clinicians’ preferred therapy in practice could shed light on how clinicians conceive of, and treat, narcissistic pathology. For instance, compared to the DSM-5 which emphasizes grandiosity, other diagnostic manuals such as the PDM-2 (Lingiardi and McWilliams, 2017) originate from psychodynamic conceptualizations of NPD which captures both grandiose and vulnerable features. This accentuates the importance of investigating the extent to which therapy modality affects diagnostic outcome.

## The present study

The current study extends the literature through exploring the implications of clinicians’ perceptions of pathological narcissism in clinical case vignettes, particularly in hypothetical female patients. Specifically, this study aims to investigate the PD diagnoses commonly leveled at hypothetical patients who present vulnerable narcissism traits, and whether clinician and patient gender play a role. The study also examines the extent to which clinicians’ preferred therapy approach and length of experience in practice influences the likelihood of diagnosis in cases with vulnerable narcissism symptomatology. Vignettes depicting grandiose features of narcissism will also be included to further explore gender differences in clinical diagnosis of NPD. These factors are explored in an online, vignette-based study with trainee and practicing clinicians.

To the knowledge of the author, this is the first study to explore clinicians’ diagnostic ratings of cases with vulnerable narcissism symptoms. As such, the present study is designed as an exploratory step toward building a cohesive and coherent understanding of the assessment, diagnosis, and treatment of narcissism, particularly in women.

## Research questions

(1). What are the common diagnostic labels given by clinicians to hypothetical patients who present symptoms of vulnerable narcissism?

(2). To what extent do clinician and patient gender influence clinicians’ diagnostic labels for cases with vulnerable narcissism symptomatology?

(3). To what extent do clinicians’ psychological therapy practices and years of experience influence diagnostic labels for cases with vulnerable narcissism symptomatology?

The current study is largely exploratory in nature and therefore offers no specific hypotheses except for the following:

*Hypothesis 1*: When presented with a vulnerable narcissism vignette depicting a female patient, clinicians will provide significantly higher diagnostic ratings of borderline, dependent, and avoidant personality disorders compared to other DSM personality disorders. This assumption is based on previous research demonstrating an overlap between vulnerable narcissism and BPD (e.g., Miller and Campbell, 2008; Euler et al., 2018), avoidant and dependent PDs (Dickinson and Pincus, 2003; Miller et al., 2014), and the observed gender bias pertaining to the overrepresentation of females in borderline and dependent PD diagnoses (American Psychiatric Association, 2013; Paris et al., 2013; Euler et al., 2018).

## Materials and methods

### Design and participants

This vignette study adopted a mixed experimental design. Independent variables were patient and clinician gender (male/female) and therapy approach (CBT/psychodynamic). The dependent variable was the likelihood of diagnosis given across a range of possible conditions. Correlational design was also employed to investigate the relationship between clinicians’ length of experience in practice and their likelihood of diagnosis given.

From the initial sample pool (*n* = 197), 89 participants were excluded due to incomplete data. The final analysis was conducted using the remaining 108 participants. The sample comprised 79 females (73.1%) and 29 males (26.9%). The age range of participants was 22–61 years with a mean of 38.31 years (SD = 9.9).

Inclusion criteria were being over 18 years of age, being fluent in English, and either having undertaken clinical practice or being active in clinical practice. Participants were predominantly Caucasian (*n* = 101), with three identified as South or East Asians, one identified as Middle Eastern, and the remaining two participants chose “mixed” or “other” for their ethnic status. Participants’ most recent qualifications were the following: Doctorate in Clinical Psychology (*n* = 35), MSc degree in Clinical Psychology/Trainee Clinical Psychologist (*n* = 17), Chartered Psychologist (*n* = 16), and Licensed Psychotherapist (*n* = 14). The remaining 26 participants did not indicate their qualifications, or their answers were ambiguous (*n* = 3).

Additional descriptive information regarding clinicians’ length of experience in practice and current psychological therapy used in practice are displayed in Table 1.

**TABLE 1:** Participant demographics.

	Total (*N* = *108)*	Males (*N* = 29)	Females (*N* = 79)
Median length of experience in months	81	138	73
Current therapy used in practice			
Cognitive behavioral therapy	61	9	52
Psychodynamic psychotherapy	13	6	7
Interpersonal therapy	4	1	3
Mindfulness-based cognitive therapy	3	1	2
Counseling	1	–	1
“Other”	26	12	14

Dashes indicate no response.

### Materials

#### Clinical case vignettes

The study used clinical case vignettes of hypothetical patients presenting prototypical expressions of vulnerable narcissism, grandiose narcissism, or panic disorder without personality pathology. The panic disorder vignette was utilized as a “distractor” condition to avoid priming clinicians toward any potential bias with regard to the aims of the current study (i.e., gender bias in personality disorders). For these purposes, the panic disorder vignette was not included in the main analyses of the current study.

The two narcissism vignettes and the panic disorder vignette were constructed by Kealy et al. (2017). The narcissism vignettes were informed by the review of Cain et al. (2008). Each vignette contained one hypothetical patient (with two versions: male and female), creating six different vignettes. Despite some male and female vignettes differing in line with gender role specific aspects, no significant clinical differences existed between them.

The research team and three highly experienced clinicians in the field of pathological narcissism and personality disorder reviewed these vignettes, which resulted in the following amendments: the male and female prototypes in the grandiose narcissism vignettes were markedly different in context, and in order to ensure consistency across all vignettes, one version of the vignette was used (with the gender inverted, thus creating male and female prototypes with identical context). The vulnerable narcissism and panic disorder vignettes were retained in their original versions.

#### Procedure

An online study (using Qualtrics) was advertised via social network sites and e-mails were sent to clinical psychology committees and organizations to distribute the study to a broader sample of clinical psychologists. After giving informed consent, participants completed demographic questions and were then randomly assigned either three male vignettes or three female vignettes to avoid priming participants to gender bias.

Participants were presented with the three vignettes: vulnerable narcissism, grandiose narcissism, and panic disorder without personality pathology. After reading each vignette, participants indicated the likelihood of diagnosis for a range of personality disorders (PDs) on a 1 (very unlikely) to 8 (very likely) rating scale, on the basis of the available history. All the PDs in the DSM-5 were listed, in order to avoid priming participants to a particular diagnosis: paranoid PD, narcissistic PD, schizoid PD, antisocial PD, borderline PD, histrionic PD, avoidant PD, dependent PD, and obsessive-compulsive PD. The choice of “other” was included based on the following reason: first, given that vulnerable narcissism is not a separate PD diagnosis in the DSM-5, clinicians had the opportunity to elaborate their justification for classifying vulnerable narcissism, or any of the other vignettes presented, as a condition separate from the PDs listed. In cases where clinicians classified vulnerable narcissism as narcissistic PD, this was interpreted as clinicians perceiving narcissistic PD as being a condition that manifests both grandiose and vulnerable traits (see Ackerman et al., 2017), despite the emphasis on grandiosity in the DSM-5 classification of NPD. After rating the vignettes, participants were debriefed and thanked for their time.

## Results

### Preliminary analyses

Preliminary analyses indicated violations of normality for the majority of variables, and data transformation did not correct the non-normality of data; thus, non-parametric tests were used. Due to multiple comparisons and tests being conducted, Type I error was controlled by a stricter alpha level of 0.01 for those cases where a Bonferroni correction had not already been applied.

**FIGURE 1:**
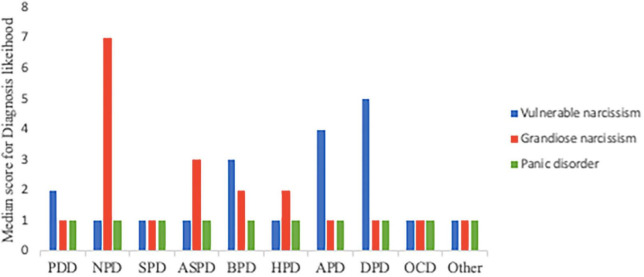
Clinicians’ likelihood of diagnosis across three conditions. PPD, paranoid PD; NPD, narcissistic PD; SPD, schizoid PD; ASPD, antisocial PD; BPD, borderline PD; HPD, histrionic PD; APD, avoidant PD; DPD, dependent PD; OCD, obsessive-compulsive PD.

Prior to exploring gender differences in diagnoses with vulnerable narcissism symptomatology, descriptive analyses were run for all vignettes to investigate the diagnoses commonly leveled at symptoms of vulnerable narcissism and grandiose narcissism. As seen in Figure 1, the most frequently endorsed diagnoses to cases with vulnerable narcissism symptomatology were dependent, avoidant, and borderline PDs (as indicated by the median score). For cases with narcissistic PD symptomatology, Figure 1 show the preferred diagnosis of NPD as indicated by the median score.^2^ As expected, clinicians’ median score was one across all PDs for the panic disorder without personality pathology vignette.

### Endorsement of diagnostic labels

Friedman’s repeated samples test was used to determine if there were any differences in the rating of the available diagnostic labels for the vulnerable narcissism vignette. The likelihood of diagnosis across conditions was the outcome variable, and the diagnostic label was entered as the independent variable. Tables 2, 3 shows the Friedman’s repeated samples test for the vulnerable narcissism vignette in male and female clinicians, respectively.

**TABLE 2:** Male clinicians’ likelihood of diagnosis in cases with vulnerable narcissism symptomatology.

	PPD	NPD	SPD	ASPD	BPD	HPD	APD	DPD	OCD	Other
Mean rank	5.45	4.91	4.31	3.41	6.60	4.91	7.76	7.91	5.19	4.53
PPD										
NPD	0.672									
SPD	1.431	0.759								
ASPD	2.559	1.887	1.128							
BPD	−1.453	−2.125	−2.884	−4.012***						
HPD	0.672	0.000	−0.759	−1.887	2.125					
APD	−2.906	−3.578*	−4.337***	−5.465***	−1.453	−3.578*				
DPD	−3.101	−3.773**	−4.532***	−5.660***	−1.648	−3.773**	−1.95			
OCD	0.325	−0.347	−1.106	−2.234	1.778	−0.347	3.231	3.426*		
Other	1.149	0.477	−0.282	−1.409	2.602	0.477	4.055***	4.250***	0.824	

Values in the lower part of the table present the test statistic = χ2. PPD, paranoid PD; NPD, narcissistic PD; SPD, schizoid PD; ASPD, antisocial PD; BPD, borderline PD; HPD, histrionic PD; APD, avoidant PD; DPD, dependent PD; OCD, obsessive-compulsive PD. *p < 0.05. **p < 0.01. ***p < 0.001.

**TABLE 3:** Female clinicians’ likelihood of diagnosis in cases with vulnerable narcissism symptomatology.

	PPD	NPD	SPD	ASPD	BPD	HPD	APD	DPD	OCD	Other
Mean rank	6.08	4.66	4.72	3.62	7.23	4.51	7.73	7.73	4.78	3.94
PPD										
NPD	2.956									
SPD	2.838	−0.118								
ASPD	5.111***	2.155	2.273							
BPD	−2.391	−5.347***	−5.229***	−7.502***						
HPD	3.258	0.302	0.420	−1.852	5.649***					
APD	−3.429*	−6.385***	−2.267***	−8.540***	−1.038	−6.687***				
DPD	−3.416*	−6.372***	−6.254***	−8.527***	−1.025	−6.674***	0.013			
OCD	2.706	−0.250	−0.131	−2.404	5.098***	−0.552	6.136***	6.122***		
Other	4.454***	1.498	1.616	−0.657	6.845***	1.196	7.883***	7.870***	1.747	

Values in the lower part of the table present the test statistic = χ2. PPD, paranoid PD; NPD, narcissistic PD; SPD, schizoid PD; ASPD, antisocial PD; BPD, borderline PD; HPD, histrionic PD; APD, avoidant PD; DPD, dependent PD; OCD, obsessive-compulsive PD. *p < 0.05. ***p < 0.001.

The Friedman test indicated a significant difference between the diagnoses attributed in the vulnerable narcissism vignette condition, for male clinicians: χ*^2^*(9) = 80.297, *p* < 0.001, and for female clinicians: χ*^2^*(9) = 266.793, *p* < 0.001. Dunn’s pairwise *post hoc* test with a Bonferroni correction applied showed that both male and female clinicians were significantly more likely to endorse the label of borderline PD compared to antisocial PD when presented with a vulnerable narcissism vignette. Male and female clinicians’ diagnosis of dependent PD was also significantly more likely endorsed compared to antisocial PD, schizoid PD, “other,” narcissistic PD, histrionic PD, and obsessive-compulsive disorder. Further, avoidant PD was significantly more likely diagnosed compared to antisocial PD, schizoid PD, “other,” narcissistic PD, and histrionic PD, for both male and female clinicians.

### Clinicians gender bias in diagnoses for cases with vulnerable narcissism symptomatology

To investigate potential gender bias in diagnoses of cases with vulnerable narcissism symptomology, a Kruskal-Wallis test was conducted to explore whether there were differences in likelihood of diagnosis between the four groups: male clinician/male patient, male clinician/female patient, female clinician/male patient, and female clinician/female patient (see Table 4). The mean ranks were compared, rather than the medians, given that the distributions in each group were not the same as indicated by visual inspection of histograms and the Levene’s test.

**TABLE 4:** Clinicians’ gender bias in diagnoses for cases with vulnerable narcissism symptomatology.

		Male C/ Male P (A)	Male C/ Female P (B)	Female C/ Male P (C)	Female C/ Female P (D)	
PD diagnosis	χ^2^	Mean rank				Pairwise comparisons
PPD	0.543	54.77	50.88	57.14	53.64	–
NPD	4.925	68.92	55.53	54.31	50.01	–
SPD	2.036	49.42	60.38	56.90	51.95	–
ASPD	4.093	56.15	58.38	57.26	50.41	–
BPD	4.618	48.35	59.16	47.09	60.52	–
HPD	7.199	52.92	70.31	49.67	53.06	–
APD	0.527	57.42	49.84	54.29	55.50	–
DPD	2.459	52.12	60.56	48.56	57.73	–
OCD	10.216*	52.62	71.75	45.43	56.00	C < B*
Other	17.216***	74.31	53.12	47.63	54.61	C < A*** D < A** B < A*

C, clinician; P, patient; PPD, paranoid PD; NPD, narcissistic PD; SPD, schizoid PD; ASPD, antisocial PD; BPD, borderline PD; HPD, histrionic PD; APD, avoidant PD; DPD, dependent PD; OCD, obsessive-compulsive PD. Dashes indicate no significant difference between groups. *p < 0.05, **p < 0.01, ***p < 0.001.

The Kruskal–Wallis test showed a significant difference between the groups for the diagnosis of “other.” Dunn-Bonferroni *post hoc* pairwise comparisons indicated that male clinicians were significantly more likely to diagnose a male patient with vulnerable symptoms as “other,” compared to all other clinician/patient gender combinations. The results pertaining to the diagnosis of “other” were followed up with *post hoc* Mann–Whitney comparisons (see Tables 5, 6). As shown in Table 5, male clinicians were significantly more likely to attribute a diagnosis of “other” and Obsessive compulsive disorder (OCD) when presented with a vulnerable narcissism vignette, compared to female clinicians. With regard to patient gender, Table 6 shows that female patients were significantly more likely to be diagnosed as BPD and OCD, compared to male patients in the vulnerable narcissism condition.

**TABLE 5:** Mann–Whitney comparisons for participant gender in vulnerable narcissism condition.

	Male clinicians (*n* = 29)	Female clinicians (*n* = 79)				
PD diagnosis	Mean rank		*U*	*z*	*p*	*r*
PPD	52.62	55.19	1091.0	0.394	0.693	0.03
NPD	61.53	51.92	941.5	−1.636	0.102	−0.15
SPD	55.47	54.15	1117.5	−0.234	0.815	−0.02
ASPD	57.38	53.44	1062.0	−1.026	0.305	−0.09
BPD	54.31	54.57	1140.0	0.039	0.969	0.00
HPD	62.52	51.56	913.0	−1.927	0.054	−0.18
APD	53.24	54.96	1109.0	0.256	0.798	0.02
DPD	56.78	53.66	1079.5	−0.463	0.643	−0.04
OCD	63.17	51.32	894.0	−1.978	0.048*	−0.19
Other	62.62	51.52	910.0	−2.576	0.010**	−0.24

PPD, paranoid PD; NPD, narcissistic PD; SPD, schizoid PD; ASPD, antisocial PD; BPD, borderline PD; HPD, histrionic PD; APD, avoidant PD; DPD, dependent PD; OCD, obsessive-compulsive PD. *p < 0.05, **p < 0.01.

**TABLE 6:** Mann–Whitney comparisons for patient gender in vulnerable narcissism condition.

	Male patients (*n* = 48)	Female patients (*n* = 60)				
PD diagnosis	Mean rank		*U*	*z*	*p*	*r*
PPD	56.50	52.90	1344.0	−0.620	0.535	−0.05
NPD	58.27	51.48	1259.0	−1.295	0.195	−0.12
SPD	54.88	54.20	1422.0	−0.134	0.893	−0.01
ASPD	56.96	52.53	1322.0	−1.293	0.196	−0.12
BPD	47.43	60.16	1100.5	2.140	0.032*	0.20
HPD	50.55	57.66	1250.5	1.401	0.161	0.13
APD	55.14	53.99	1409.5	−0.191	0.848	−0.01
DPD	49.52	58.48	1201.0	1.495	0.135	0.14
OCD	47.38	60.20	1098.0	2.399	0.016*	0.23
Other	54.85	54.22	1423.0	−0.166	0.868	−0.01

PPD, paranoid PD; NPD, narcissistic PD; SPD, schizoid PD; ASPD, antisocial PD; BPD, borderline PD; HPD, histrionic PD; APD, avoidant PD; DPD, dependent PD; OCD, obsessive-compulsive PD. *p < 0.05.

### Clinicians’ psychological therapy and years of experience

In order to investigate differences between clinicians’ main psychological therapeutic approach in practice, two groups were created based on the underlying conceptual foundation for their therapy: “Psychodynamic” (including participants who identified Psychodynamic psychotherapy or Interpersonal therapy as their main therapeutic approach) and “CBT” (including CBT and Mindfulness-based cognitive therapy); participants who selected other therapeutic approaches were not included (due to limited group sizes). Mann–Whitney tests were conducted to explore differences between clinicians’ underlying therapeutic approach and the likelihood of diagnosis given in the vulnerable narcissism condition (see Table 7). Clinicians with a psychodynamic approach were significantly more likely to diagnose vulnerable narcissism as narcissistic PD compared to those with a CBT approach. Clinicians with a psychodynamic approach were also significantly more likely to diagnose vulnerable narcissism as obsessive-compulsive disorder, compared to those with a CBT approach.

**TABLE 7:** Mann–Whitney comparisons for clinicians’ therapy modalities in vulnerable narcissism condition.

	Psychodynamic (*n* = 17)	CBT (*n* = 64)				
PD diagnosis	Mean rank		*U*	*z*	*p*	*r*
PPD	48.00	39.14	425.0	−1.434	0.151	−0.15
NPD	52.47	37.95	349.0	−2.771	0.006**	−0.30
SPD	41.65	40.83	533.0	−0.158	0.874	−0.01
ASPD	43.82	40.25	496.0	−1.022	0.307	−0.11
BPD	44.79	39.99	479.5	−0.763	0.445	−0.08
HPD	42.82	40.52	513.0	−0.425	0.671	−0.04
APD	48.09	39.12	423.5	−1.416	0.157	−0.15
DPD	45.18	39.89	473.0	−0.834	0.405	−0.09
OCD	54.15	37.51	320.5	−2.904	0.004**	−0.32
Other	36.71	42.14	471.0	−1.325	0.185	−0.14

PPD, paranoid PD; NPD, narcissistic PD; SPD, schizoid PD; ASPD, antisocial PD; BPD, borderline PD; HPD, histrionic PD; APD, avoidant PD; DPD, dependent PD; OCD, obsessive-compulsive PD. **p < 0.01.

A Mann–Whitney test was conducted to explore these patterns in males and females separately. The only significant difference was found for female clinicians: females with a psychodynamic approach were significantly more likely to diagnose vulnerable narcissism as narcissistic PD compared to those with a CBT approach (see Supplementary material for these data).

Spearman’s rho was conducted to explore correlations between clinicians’ length of experience and the likelihood of the particular diagnosis given (see Table 8). Interestingly, length of experience was positively significantly correlated with attributing narcissistic PD diagnosis when presented with symptoms of vulnerable narcissism vignette. Conducting these separately for male and female clinicians revealed that this finding was only significant in females.

**TABLE 8:** Spearman’s rho correlations in clinicians between length of experience and diagnosis in vulnerable narcissism condition.

	Clinicians (*n* = 108)	Males clinicians (*n* = 29)	Females clinicians (*n* = 79)
**PD diagnosis**	**Length of experience**		
PPD	0.043	0.224	0.004
NPD	0.304**	0.153	0.332**
SPD	0.071	0.253	0.016
ASPD	0.050	0.199	−0.043
BPD	0.084	0.181	0.046
HPD	0.035	0.052	−0.003
APD	0.069	0.056	0.061
DPD	0.013	−0.059	0.023
OCD	0.067	0.094	0.041
Other	0.051	0.121	−0.003

PPD, paranoid PD; NPD, narcissistic PD; SPD, schizoid PD; ASPD, antisocial PD; BPD, borderline PD; HPD, histrionic PD; APD, avoidant PD; DPD, dependent PD; OCD, obsessive-compulsive PD. **p < 0.01.

## Discussion

The purpose of the current study was to explore the extent to which clinicians influence the diagnostic labels commonly attributed to cases with vulnerable narcissism symptomatology, particularly in hypothetical female patients. The role of clinicians’ gender, therapeutic orientations, and length of experience were examined given the potential they could influence such diagnostic labels on the one hand, and their relevance to the assessment and treatment of pathological narcissism in women on the other.

The hypothesis that the borderline, dependent, and avoidant PD diagnoses are most frequently endorsed when clinicians are presented with a female patient exhibiting vulnerable narcissism symptoms, compared to other DSM personality disorders, was supported. These findings resonate with previous research demonstrating an overlap between vulnerable narcissism and borderline PD (Miller and Campbell, 2008; Euler et al., 2018), and avoidant and dependent PD (Dickinson and Pincus, 2003; Miller et al., 2014). More importantly, the findings of this study also showed that, when presented with a vulnerable narcissism vignette, clinicians were significantly more likely to attribute a BPD diagnosis in female patients, compared to male patients. The current results provide implications for gender bias in the DSM in general, and for the assessment and treatment of vulnerable narcissism in particular. With regard to the former, the current findings suggest that the observed gender bias pertaining to the overrepresentation of females in borderline and dependent PD diagnoses (American Psychiatric Association, 2013; Paris et al., 2013; Euler et al., 2018) may, in part, be attributed to how clinicians perceive narcissistic vulnerability symptoms in female patients. This is particularly significant considering previous research suggesting that females are more likely to seek treatment than males (Skodol and Bender, 2003), and clinicians are more likely to treat patients who present narcissistic pathology of the vulnerable type (Ellison et al., 2013), features which tend to be more prevalent in narcissistic females (Green et al., 2021).

When presented with symptoms of vulnerable narcissism, male clinicians were significantly more likely to diagnose a male patient as “other” (e.g., social anxiety and depression), compared to all other clinician/patient gender combinations, and this process appeared to be influenced by clinicians’ gender rather than patient gender which was further indicated by the follow-up Mann–Whitney analyses. This gender difference in clinicians is particularly interesting, especially considering that the provision of differential rates of diagnosis has traditionally been understood to be the result of clinicians assigning different diagnoses based on patient’s gender. Indeed, there have only been isolated findings of clinician gender affecting diagnosis (Crosby and Sprock, 2004).

Nevertheless, the clinician gender difference found in this study can be interpreted in numerous ways. These findings may indicate the potential of gender stereotyping on part of the clinician, given the fact that male clinicians were more likely to apply sets of symptoms to male patients, whereas female patients were diagnosed differently despite exhibiting identical symptomatology. It can therefore be conjectured that male clinicians may perceive the same symptoms differently depending on the patient’s gender and the concomitant gender weighting of the symptoms. Narcissistic vulnerability symptoms overlap with many “typically feminine” disorders (e.g., BPD and DPD), and thus might account for clinicians’ diagnostic bias toward categorization of female but not male patients. This would resonate with Flanagan and Blashfield’s (2003) study, showing that when participants are taught gender associations with the personality disorder categories, they are more likely to rate the personality disorder cases in accordance with those associations (e.g., BPD associated with females and ASPD associated with males).

Moreover, despite the overwhelming evidence that grandiose narcissism (NPD DSM) appears to be diagnosed more often in males than in females, the results of this study showed clinicians were attributing the diagnosis in a gender-neutral fashion. This finding is less consistent with theoretical speculation that clinicians are gender biased in application of diagnostic sets in relation to male patients (e.g., Anderson et al., 2001). It could be argued that the differential prevalence rates within males and females diagnosed with NPD in the DSM-5 may simply be an artifact of actual sex differences, where males are more likely to present features of grandiose narcissism compared to females (Euler et al., 2018). These findings are important considering the criticism that may be leveled at the current aims of this study—investigating gender bias in a condition that is inherently gender-biased. Instead, what these findings show is that, at least for clinicians, their understanding of NPD is not necessarily that it is exclusively a male pathology. However, it also needs to be acknowledged that the discrepancy between the findings here and previous research may be partly due to differences in diagnostic criteria and assessment instruments.

Results further showed that clinicians with a psychodynamic orientation, but not CBT, were significantly more likely to diagnose vulnerable narcissism as NPD. It is not surprising that a psychodynamic approach would recognize vulnerable features of narcissism in its theoretical formulations. Psychodynamic approaches tend to emphasize personality development, relational and intrapsychic dynamics which are guided by the work of, among others, Kernberg (1975) and Kohut (1977). CBT clinicians, on the other hand, are more rigid in the sense that they tend to focus entirely, if not exclusively, on immediate symptoms and cognitions rather than on the concept of personality (Hofmann and Hayes, 2019). In terms of clinical implications, these findings provide more credence to other diagnostic manuals that contain a more comprehensive diagnostic definition of NPD, spanning grandiose and vulnerable features (e.g., PDM-2; Lingiardi and McWilliams, 2017).

Nevertheless, the finding that a clinician’s theoretical orientation affects their diagnostic judgment has an impact on how patients are assessed, the treatment plans constructed, and possibly the effectiveness of such interventions. Future research should replicate and explore these patterns using more established manuals such as the PDM-2 and the ICD-11. The results of this study also showed that the more experience a clinician had, the more likely they were to attribute vulnerable narcissism as being NPD. It is stressed here that the DSM-5 diagnostic procedure as it currently stands is questionable in its suitability for purpose, as clinicians are only able to diagnose vulnerable narcissism as NPD once they have gained experience in the differences between NPD as captured in the DSM nomenclature and the psychiatric phenomenon that they observe of narcissism in practice.

### Limitations and future directions

Given the vignette-based design of this study, it is difficult to determine the extent to which current results can be generalized to actual clinician-patient interactions and diagnostic interviews. One clinician even declined to partake in this study on the grounds that they considered it unethical to provide a personality diagnosis based on a short description of a patient vignette. Therefore, it is arguable that a limitation of this study is that the use of clinical case vignettes, and not actual patients, may have influenced the external validity of the study. In addition, although the sample size (*n* = 108) is comparable with prior research in this field, the relatively modest sample size between the groups (29 males), may have been underpowered to detect differences. Nevertheless, it was possible to identify a number of significant differences were obtained despite these limitations.

In terms of suggestions for future directions, it would be of interest to explore whether gender differences occur or are diminished according to which particular symptoms are displayed in vignettes. Such data may allow for the delineation of specific symptoms which impact on the presentation of narcissism in males and females, and thus may require gender-sensitive interventions that address such indicators. Future research should also explore gender differences in patients with narcissistic pathology to evaluate whether expressions of narcissism shift depending on the severity of dysfunction (Kealy et al., 2016).

Future research should also consider exploring gender bias in narcissistic pathology using dimensional ratings derived from a Five Factor Model of personality (FFM; Widiger and Costa, 2002), such as the ICD-11, Personality Inventory for DSM-5 (PID-5; Krueger et al., 2014) or the Five-Factor Narcissism Inventory (FFNI; Miller et al., 2013). Moreover, in line with growing evidence that individuals fluctuate between grandiose and vulnerable states (e.g., Pincus et al., 2009; Edershile et al., 2019; Edershile and Wright, 2021), future research should explore clinician perception of narcissistic pathology in women across different state measures (e.g., EMA, Ecological Momentary Assessment) and the extent to which these perceptions influence diagnostic conceptualization. Such foci would complement the findings of the current study and expand theoretical knowledge regarding gender disparities in narcissistic presentations. Finally, the current study could be replicated to explore whether a clinician’s own gender role attributes gender bias when responding to a patient’s symptoms. This is particularly noteworthy considering previous research showing bias in the application of personality diagnosis, with symptoms that were inconsistent with a clinician’s gender role being viewed as more pathological in contrast to symptoms that were consistent with clinician’s gender role viewed as being less pathological (Crosby and Sprock, 2004).

Overall, the results of this study contribute novel knowledge of how clinicians perceive pathological narcissism in females, through identifying characteristics on the part of the clinician that influence likelihood of diagnosis in cases with vulnerable narcissism symptomatology. These findings ultimately pose challenges to the theoretical and clinical utility of NPD captured in the nosological system in relation to gender, the differential prevalence rates among males and females, and the overlap of vulnerable narcissism with other personality disorders. The clinical implications of these findings accentuate the growing recognition of the limitations in the assessments of personality disorders as discrete clinical conditions (see Hopwood et al., 2018).

## Data availability statement

The raw data supporting the conclusions of this article will be made available by the authors, without undue reservation.

## Ethics statement

The studies involving human participants were reviewed and approved by the Edinburgh Napier University Research Ethics Committee. The participants provided their written informed consent to participate in this study.

## Author contributions

AG was responsible for the conception and design of the study, data collection, performed the statistical analyses, and wrote the first draft of the manuscript. RM contributed to the design, statistical analyses, and reviewed and edited the manuscript. KC contributed to the design and reviewed and edited the manuscript. All authors contributed to the article and approved the submitted version.

## Footnotes

1 Although the current study focuses on gender differences, we have used the terms “sex bias” and “gender bias” in this paper to reflect the language adopted by the original authors, and although these constructs are similar, we acknowledge that they are distinct and not necessarily interchangeable.

2 Due to the journal’s word restrictions, subsequent analyses on NPD are included in Supplementary material.
