# Supplementary material for: Clinician perception of pathological narcissism in females: a vignette-based study
Source: Front Psychol. 2023 Apr 20;14:1090746. doi: 10.3389/fpsyg.2023.1090746 (PMC10157482; doi:10.3389/fpsyg.2023.1090746)
Supplement: Supplementary file 1 [file Table_1.docx]

Supplementary materials

| Table 1.  *Male clinicians’ likelihood of diagnosis in cases with grandiose narcissism symptomatology* | | | | | | | | | | | |
| --- | --- | --- | --- | --- | --- | --- | --- | --- | --- | --- | --- |
|  | PPD | NPD | SPD | ASPD | BPD | HPD | APD | DPD | OCD | Other |  |
| Mean rank | 4.88 | 9.67 | 4.38 | 6.86 | 6.24 | 5.79 | 3.83 | 3.95 | 5.07 | 4.33 |  |
| PPD |  |  |  |  |  |  |  |  |  |  |  |
| NPD | -6.028*** |  |  |  |  |  |  |  |  |  |  |
| SPD | .629 | -6.657*** |  |  |  |  |  |  |  |  |  |
| ASPD | -2.494 | 3.535* | -3.123 |  |  |  |  |  |  |  |  |
| BPD | -1.713 | 4.315*** | -2.342 | .781 |  |  |  |  |  |  |  |
| HPD | -1.149 | 4.879*** | -1.778 | 1.344 | .564 |  |  |  |  |  |  |
| APD | 1.323 | 7.351*** | .694 | 3.816** | 3.036 | 2.472 |  |  |  |  |  |
| DPD | 1.171 | 7.199*** | .542 | 3.665* | 2.884 | 2.320 | -.152 |  |  |  |  |
| OCD | -.239 | 5.790*** | -.867 | 2.255 | 1.475 | .911 | -1.561 | -1.409 |  |  |  |
| Other | .694 | 6.722*** | .065 | 3.188 | 2.407 | 1.843 | -.629 | -.477 | .932 |  |  |

*Note.* Values in the lower part of the table present the test statistic = χ2. PPD = Paranoid PD, NPD = Narcissistic PD,

SPD = Schizoid PD, ASPD = Antisocial PD, BPD = Borderline PD, HPD = Histrionic PD, APD = Avoidant PD,

DPD = Dependent PD, and OCD = Obsessive-compulsive PD. *p<0.05. **p<0.01. ***p<0.001.

| Table 2.  *Female clinicians’ likelihood of diagnosis in cases with grandiose narcissism symptomatology* | | | | | | | | | | |
| --- | --- | --- | --- | --- | --- | --- | --- | --- | --- | --- |
|  | PPD | NPD | SPD | ASPD | BPD | HPD | APD | DPD | OCD | Other |
| Mean rank | 4.95 | 9.92 | 4.28 | 7.06 | 6.41 | 6.11 | 3.97 | 3.90 | 4.60 | 3.79 |
| PPD |  |  |  |  |  |  |  |  |  |  |
| NPD | -10.313*** |  |  |  |  |  |  |  |  |  |
| SPD | 1.380 | 11.693*** |  |  |  |  |  |  |  |  |
| ASPD | -4.388*** | 5.925*** | -5.768*** |  |  |  |  |  |  |  |
| BPD | -3.035 | 7.279*** | -4.414*** | 1.353 |  |  |  |  |  |  |
| HPD | -2.404 | 7.909*** | -3.784** | 1.984 | .631 |  |  |  |  |  |
| APD | 2.023 | 12.337*** | .644 | 6.411*** | 5.058*** | 4.428*** |  |  |  |  |
| DPD | 2.181 | 12.494*** | .801 | 6.569*** | 5.216*** | 4.585*** | .158 |  |  |  |
| OCD | .723 | 11.036*** | -.657 | 5.111*** | 3.758** | 3.127 | -1.301 | -1.458 |  |  |
| Other | 2.404 | 12.718*** | 1.025 | 6.792*** | 5.439*** | 4.809*** | .381 | .223 | 1.682 |  |

*Note.* Values in the lower part of the table present the test statistic = χ2. PPD = Paranoid PD, NPD = Narcissistic PD, SPD = Schizoid PD,

ASPD = Antisocial PD, BPD = Borderline PD, HPD = Histrionic PD, APD = Avoidant PD, DPD = Dependent PD, and OCD = Obsessive-compulsive PD.

**p<0.01. ***p<0.001.

| Table 3.  *Clinicians’ gender bias in diagnoses for cases with grandiose narcissism symptomatology* | | | | | | |
| --- | --- | --- | --- | --- | --- | --- |
|  | | Male C/ Male P (A) | Male C/ Female P  (B) | Female C/ Male P  (C) | Female C/ Female P  (D) |  |
| PD diagnosis | *χ*^2^ | Mean rank | | | | Pairwise comparisons |
| PPD | .441 | 58.08 | 51.34 | 54.87 | 54.30 | - |
| NPD | 5.027 | 66.35 | 55.03 | 58.30 | 47.78 | - |
| SPD | .560 | 54.69 | 56.00 | 52.16 | 55.76 | - |
| ASPD | 11.368** | 65.77 | 42.78 | 65.46 | 46.72 | D vs. C* |
| BPD | 2.507 | 47.12 | 58.38 | 50.34 | 58.58 | - |
| HPD | 4.673 | 52.23 | 51.91 | 57.56 | 61.64 | - |
| APD | 3.479 | 49.88 | 59.09 | 50.87 | 57.08 | - |
| DPD | 2.456 | 50.92 | 60.81 | 53.14 | 54.34 | - |
| OCD | 1.739 | 55.35 | 61.75 | 51.53 | 53.98 | - |
| Other | 2.952 | 61.19 | 56.06 | 53.61 | 52.66 | - |

*Note.* C = Clinician. P = Patient. PPD = Paranoid PD, NPD = Narcissistic PD, SPD = Schizoid PD, ASPD = Antisocial PD, BPD = Borderline PD, HPD = Histrionic PD, APD = Avoidant PD, DPD = Dependent PD, and OCD = Obsessive-compulsive PD. Dashes indicate no significant difference between groups. *p<0.05. **p<0.01

| Table 4.  *Mann-Whitney comparisons for participant gender in grandiose narcissism condition* | | | | | | |
| --- | --- | --- | --- | --- | --- | --- |
|  | Male  clinicians  (*n* = 29) | Female clinicians  (*n* = 79) |  | | | |
| PD Diagnosis | Mean Rank | | *U* | *z* | *p* | *r* |
| PPD | 54.36 | 54.55 | 1,149.5 | .032 | .975 | .003 |
| NPD | 60.10 | 52.44 | 983.0 | -1.204 | .229 | -.11 |
| SPD | 55.41 | 54.16 | 1,119.0 | -.249 | .803 | -.02 |
| ASPD | 53.09 | 55.02 | 1,186.5 | .290 | .772 | .02 |
| BPD | 53.33 | 54.93 | 1.179.5 | .244 | .807 | .02 |
| HPD | 52.05 | 55.40 | 1,216.5 | .520 | .603 | .05 |
| APD | 54.97 | 54.33 | 1,132.0 | -.148 | .883 | -.01 |
| DPD | 56.38 | 53.81 | 1,091.0 | -.629 | .529 | -.06 |
| OCD | 58.88 | 52.89 | 1,018.5 | -1.063 | .288 | -.10 |
| Other | 58.36 | 53.08 | 1,033.5 | -1.479 | .139 | -.14 |

*Note.* PPD = Paranoid PD, NPD = Narcissistic PD, SPD = Schizoid PD, ASPD = Antisocial PD, BPD = Borderline PD, HPD = Histrionic PD, APD = Avoidant PD, DPD = Dependent PD, and OCD = Obsessive-compulsive PD.

| Table 5.  *Mann-Whitney comparisons for patient gender in grandiose narcissism condition* | | | | | | |
| --- | --- | --- | --- | --- | --- | --- |
|  | Male patients (*n* = 48) | Female patients (*n* = 60) |  | | | |
| PD Diagnosis | Mean Rank | | *U* | *z* | *p* | *r* |
| PPD | 55.74 | 53.51 | 1,380.5 | -.420 | .675 | -.04 |
| NPD | 60.48 | 49.72 | 1,153.0 | -1.896 | .058 | -.18 |
| SPD | 52.84 | 55.82 | 1,519.5 | .667 | .505 | .06 |
| ASPD | 65.54 | 45.67 | 910.0 | -3.343 | .001*** | -.32 |
| BPD | 49.47 | 58.52 | 1,681.5 | 1.549 | .121 | .14 |
| HPD | 48.82 | 59.04 | 1,712.5 | 1.781 | .075 | .17 |
| APD | 50.60 | 57.62 | 1,627.0 | 1.826 | .068 | .17 |
| DPD | 52.54 | 56.07 | 1,534.0 | .967 | .333 | .09 |
| OCD | 52.56 | 56.05 | 1,533.0 | .694 | .488 | .06 |
| Other | 55.67 | 53.57 | 1,384.0 | -.660 | .509 | -.06 |

*Note.* PPD = Paranoid PD, NPD = Narcissistic PD, SPD = Schizoid PD, ASPD = Antisocial PD, BPD = Borderline PD, HPD = Histrionic PD, APD = Avoidant PD, DPD = Dependent PD, and OCD = Obsessive-compulsive PD. ***p<0.001.

| Table 6.  *Mann-Whitney comparisons for male clinicians’ therapy modalities in vulnerable narcissism condition* | | | | | | |
| --- | --- | --- | --- | --- | --- | --- |
|  | Psychodynamic  (*n* = 7) | CBT  (*n* = 10) |  | | | |
| PD Diagnosis | Mean Rank | | *U* | *z* | *p* | *r* |
| PPD | 8.50 | 9.35 | 38.5 | .363 | .740 | .08 |
| NPD | 9.21 | 8.85 | 33.5 | -.173 | .887 | -.04 |
| SPD | 7.57 | 10.00 | 45.0 | 1.216 | .364 | .27 |
| ASPD | 8.00 | 9.70 | 42.0 | 1.222 | .536 | .29 |
| BPD | 7.29 | 10.20 | 47.0 | 1.216 | .270 | .29 |
| HPD | 6.50 | 10.75 | 52.5 | 1.849 | .088 | .44 |
| APD | 7.43 | 10.10 | 46.0 | 1.104 | .315 | .26 |
| DPD | 6.64 | 10.65 | 51.5 | 1.652 | .109 | .40 |
| OCD | 11.07 | 7.55 | 20.5 | -1.486 | .161 | -.36 |
| Other | 7.71 | 9.90 | 44.0 | 1.091 | .417 | .26 |

*Note.* PPD = Paranoid PD, NPD = Narcissistic PD, SPD = Schizoid PD, ASPD = Antisocial PD, BPD = Borderline PD, HPD = Histrionic PD, APD = Avoidant PD, DPD = Dependent PD, and OCD = Obsessive-compulsive PD.

| Table 7.  *Mann-Whitney comparisons for female clinicians’ therapy modalities in vulnerable narcissism condition* | | | | | | |
| --- | --- | --- | --- | --- | --- | --- |
|  | Psychodynamic  (*n* = 10) | CBT  (*n* = 54) |  | | | |
| PD Diagnosis | Mean Rank | | *U* | *z* | *p* | *r* |
| PPD | 45.20 | 30.15 | 143.0 | -2.429 | .015* | -.30 |
| NPD | 46.25 | 29.95 | 132.5 | -3.153 | .002** | -.39 |
| SPD | 37.10 | 31.65 | 224.0 | -1.055 | .291 | -.13 |
| ASPD | 38.80 | 31.33 | 207.0 | -2.153 | .031* | -.26 |
| BPD | 43.55 | 30.45 | 159.5 | -2.081 | .037* | -.26 |
| HPD | 37.95 | 31.49 | 215.5 | -1.250 | .211 | -.15 |
| APD | 45.45 | 30.10 | 140.5 | -2.432 | .015* | -.30 |
| DPD | 41.50 | 30.83 | 180.0 | -1.686 | .092 | -.21 |
| OCD | 42.60 | 30.63 | 169.0 | -2.156 | .031* | -.26 |
| Other | 28.50 | 33.24 | 310.0 | 1.287 | .198 | .16 |

*Note.* PPD = Paranoid PD, NPD = Narcissistic PD, SPD = Schizoid PD, ASPD = Antisocial PD, BPD = Borderline PD, HPD = Histrionic PD, APD = Avoidant PD, DPD = Dependent PD, and OCD = Obsessive-compulsive PD. *p<0.05. **p<0.01.

| Table 8.  *Mann-Whitney comparisons for clinicians’ therapy modalities in grandiose narcissism condition* | | | | | | | |
| --- | --- | --- | --- | --- | --- | --- | --- |
|  | Psychodynamic  (*n* = 17) | CBT  (*n* = 64) |  | | | |  |
| PD Diagnosis | Mean Rank | | *U* | *z* | *p* | *r* |  |
| PPD | 43.62 | 40.30 | 499.5 | -.589 | .556 | -.06 |  |
| NPD | 44.53 | 40.06 | 484.0 | -.746 | .456 | -.08 |  |
| SPD | 35.26 | 42.52 | 641.5 | 1.632 | .103 | .18 |  |
| ASPD | 39.38 | 41.43 | 571.5 | .324 | .746 | .03 |  |
| BPD | 41.32 | 40.91 | 538.5 | -.066 | .947 | -.007 |  |
| HPD | 39.24 | 41.47 | 574.0 | .369 | .712 | .04 |  |
| APD | 42.71 | 40.55 | 515.0 | -.589 | .556 | -.06 |  |
| DPD | 45.62 | 39.77 | 465.5 | -1.594 | .111 | -.17 |  |
| OCD | 49.44 | 38.76 | 400.5 | -2.040 | .041* | -.22 |  |
| Other | 39.76 | 41.33 | 565.0 | .500 | .617 | .05 |  |

*Note.* PPD = Paranoid PD, NPD = Narcissistic PD, SPD = Schizoid PD, ASPD = Antisocial PD,

BPD = Borderline PD, HPD = Histrionic PD, APD = Avoidant PD, DPD = Dependent PD, and OCD = Obsessive-compulsive PD. *p<0.05.

| Table 9.  *Spearman’s rho correlations between length of experience and diagnosis in the grandiose narcissism condition* | | | | | | |
| --- | --- | --- | --- | --- | --- | --- |
| PD Diagnosis |  |  |  | Length of experience |  |  |
| PPD |  |  |  | .146 |  | |
| NPD | |  | | -.093 |  | |
| SPD | |  | | .031 |  | |
| ASPD | |  | | -.048 |  | |
| BPD | |  | | -.028 |  | |
| HPD | |  | | -.073 |  | |
| APD | |  | | .029 |  | |
| DPD | |  | | .086 |  | |
| OCD | |  | | .110 |  | |
| Other | |  | | .086 |  | |

*Note*. PPD = Paranoid PD, NPD = Narcissistic PD, SPD = Schizoid PD, ASPD = Antisocial PD, BPD = Borderline PD, HPD = Histrionic PD, APD = Avoidant PD, DPD = Dependent PD, and OCD = Obsessive-compulsive PD.
